# Morphological and Molecular Identification of Microcystin-Producing Cyanobacteria in Nine Shallow Bulgarian Water Bodies

**DOI:** 10.3390/toxins12010039

**Published:** 2020-01-08

**Authors:** Mariana Radkova, Katerina Stefanova, Blagoy Uzunov, Georg Gärtner, Maya Stoyneva-Gärtner

**Affiliations:** 1 AgroBio Institute, Bulgarian Agricultural Academy, BG-1164 Sofia, Bulgaria; marianaradkova@yahoo.com (M.R.); katerina_stefanova@abi.bg (K.S.); 2 Faculty of Biology, Department of Botany, Sofia University, BG-1164 Sofia, Bulgaria; mstoyneva@abv.bg; 3 Institute of Botany, Innsbruck University, A-6020 Innsbruck, Austria; georg.gaertner@uibk.ac.at

**Keywords:** cyanoprokaryotes, cyanotoxins, nodularins, phytoplankton, polyphasic approach

## Abstract

The paper presents results from the first application of polyphasic approach in studies of field samples from Bulgaria. This approach, which combined the conventional light microscopy (LM) and molecular-genetic methods (based on PCR amplified fragments of microcystin synthetase gene *mcyE*), revealed that almost all microcystin-producers in the studied eutrophic waterbodies belong to the genus *Microcystis*. During the molecular identification of toxin-producing strains by use of HEPF × HEPR pair of primers, we obtained 57 sequences, 56 of which formed 28 strains of *Microcystis,* spread in six clusters of the phylogenetic tree. By LM, seven *Microcystis* morphospecies were identified (*M. aeruginosa*, *M. botrys*, *M. flos*-*aquae, M. natans*, *M. novacekii, M. smithii*, and *M. wesenbergii*). They showed significant morphological variability and contributed from <1% to 98% to the total biomass. All data support the earlier opinions that taxonomic revision of *Microcystis* is needed*,* proved the presence of toxigenic strains in *M. aeruginosa* and *M. wesenbergii*, and suppose their existence in *M. natans*. Our results demonstrated also that genetic sequencing, and the use of HEPF × HEPR pair in particular, can efficiently serve in water quality monitoring for identifying the potential risk from microcystins, even in cases of low amounts of *Microcystis* in the water.

## 1. Introduction

Some hazardous toxigenic species from the phylum Cyanoprokaryota/Cyanobacteria (known also as blue-green algae) can form harmful algal blooms commonly abbreviated as CyanoHABs. The increasing and widespread concern regarding the serious threat which these algae and their toxins (cyanotoxins) pose to human and animal health is consistent with the recent growth in interest on the topic [1]. In Bulgaria, from 120 water bodies (WBs) investigated during the period 2000–2015, cyanoprokaryotic blooms were recorded in 14 WBs, and in 16 WBs cyanotoxins (mostly microcystins—MCs, but also nodularins—NODs and saxitoxins—SXTs) were found [2,3]. In a more recent study, conducted in 2018, a CyanoHab with MCs was detected in one more Bulgarian inland reservoir, namely Sinyata Reka [4]. From the samples with detected cyanotoxins in the period 2000–2015, 44 cyanoprokaryotes were determined by light microscopy (LM) [2]. However, it is widely known that the traditional LM identification of some bloom causative species remains problematic because of the unresolved taxonomy of certain cyanoprokaryote genera. The phenotypic flexibility of some standard diagnostic features in the taxonomy of cyanoprokaryotes—including the shape and structure of colonies, presence/absence of gas vesicles, akinetes, or heterocytes [5,6,7,8]—has promoted the parallel use of both morphological and molecular phylogenetic data in a common polyphasic approach [9,10,11]. This approach includes different molecular techniques and is increasingly employed for the analysis of environmental samples (e.g., [12,13,14,15,16,17,18,19]). However, the assignment of taxa to sequences is often a challenge in molecular-based classification methods applied to environmental samples [19,20]. For early detection of potentially toxigenic cyanobacteria and cyanotoxins, rapid, reliable, and economically effective molecular approaches based on a PCR (polymerase chain reaction) targeting genes involved in the biosynthesis of cyanotoxins have been developed in the past 15 years [21]. Cyanotoxins which include the group of potent hepatotoxins—MCs and NODs—are produced through non-ribosomal peptide synthesis and exhibit biosynthetic routes that shared many similarities [22]. For MCs in particular, the large enzyme complex involved in their production is encoded by a cluster of 10 genes, called *microcystin synthetase genes* (*mcyA-J*) [22], from which the NOD gene cluster (*nda* cluster) arose [23]. The identification of MC-producing species and strains through amplifications of six of these genes (*mcy A-E*) became most commonly applied [21,24]. Due to the absence of *mcy* genes in non-toxigenic species or strains, standard PCR with relevant *mcy*-specific primers provides a quick qualitative tool to discriminate between potentially toxic and nontoxic algae in the water monitoring and general ecological studies [21,25,26]. To get information about actual toxin concentrations through molecular tools, efforts were made to correlate cyanotoxin biosynthesis gene abundance with cyanotoxin occurrence and concentrations (for details see [27]). Up to now, these tools were most successful for MCs and NODs [27] and for the estimation of cyanotoxin gene abundance in field populations, a quantitative PCR (*qPCR*, or *real-time PCR*) was developed [21].

Early detection of toxic cyanobacteria by real-time PCR was already successfully applied in Bulgaria [28] but was not combined with LM studies. Therefore, the aim of the present paper is to compare the results from recent LM observations and PCR-based molecular identification of MC- and NOD-producing cyanoprokaryotes in field samples from nine shallow WBs of Bulgaria. Both MCs (together with their close NODs) and WBs were chosen according to the results from previous research, which indicated the threat of CyanoHabs [2,3]. Considering the most common ways of potential exposure of humans to cyanotoxins through consumption of unsuitably treated drinking water, through recreational activities, or through consumption of fish, mollusks and crayfish (e.g., [27,29,30]), it has to be underlined that all chosen WBs are used for recreation and sport fishing, and four of them are dams for water supply and irrigation [2,31].

The results obtained during the study indicated the MC risk on studied WBs caused by the presence of 28 MC-producing strains of the genus *Microcystis,* seven species of which were confirmed by LM. The data obtained from the combination of conventional microscopic studies with PCR-sequencing: (1) proved the presence of toxic strains in *M. wesenbergii*; (2) allowed strongly to suppose their existence in *M. natans*; (3) confirmed the well-known presence of toxigenic strains in *M. aeruginosa*; (4) showed the significant morphological variability of *Microcystis* colonies with many transitional colonies, and, in combination with the complex genetic pool, supported the earlier opinions that taxonomic revision of the genus is needed, and (5) once more demonstrated that genetic sequencing, and the use of HEPF × HEPR pair of primers in particular, can efficiently serve in water quality monitoring for identifying the potential risk from MCs, even in cases of low amounts of *Microcystis* in the water. When considering the results obtained by other methods from the study of the same WBs in the same period [4], we share the widely accepted opinion that until a unique method is adopted, a combination of different approaches is more desirable, and even needed, in the studies of CyanoHABs.

## 2. Results

### 2.1. Phytoplankton Species Composition Obtained by Light Microscopy (LM)

In total, more than 240 species from different taxonomic groups were identified using LM in the phytoplankton of all studied WBs, and the reliably determined cyanoprokaryotes comprised 68, or 28% of all taxa. They belonged to 30 genera from four orders (Chroococcales, Synechococcales, Oscillatoriales, and Nostocales). The highest number of cyanoprokaryotes was found in the coastal lakes Vaya (43) and Durankulak (22), followed by the coastal reservoirs Mandra (9), Poroy (8), Aheloy (3), and inland reservoir Sinyata Reka (2) and coastal lake Uzungeren (1). Cyanoprokaryotes were not found in the coastal lakes Shabla and Ezerets.

Considering the provided below results from the PCR analysis, which showed the presence of MC-producers, but lack of NOD-producers, we would like to underline the absence in the processed samples of its main producer—the heterocytous filamentous genus *Nodularia* Mertens (e.g., [22]). Hereafter only the LM data obtained on the coccoid genus *Microcystis* Kützing ex Lemmermann will be presented with a note that species from its morphologically close genera *Aphanocapsa* Nägeli, *Coelomoron* Buell, *Coelosphaerium* Nägeli, and *Pannus* Hickel were also determined in the processed slides.

Using traditional morphological diagnostic features [32,33,34,35,36,37,38], we identified seven species of *Microcystis: M. aeruginosa* (Kützing) Kützing, *M. botrys* Teiling, *M. flos-aquae* (Wittrock) Kirchner, *M. natans* Lemmermann ex Skuja, *M. novacekii* (Komárek) Compère, *M. smithii* Komárek et Anagnostidis, and *M. wesenbergii* (Komárek) Komárek in Kondratieva 1964. In addition, in some of the samples we found separate *Microcystis* cells from disintegrated colonies, which did not allow their correct and reliable identification using classical taxonomic criteria. Within most identified species, a significant morphological variation was observed, sometimes showing transitional features with other species, or even genera.

The differences in the distribution of *Microcystis* species by WBs and sites are shown in Table 1. Their quantitative role was very low, <1% of the total phytoplankton biomass except their high contribution (99.8%) in the small inland reservoir Sinyata Reka.

**Figure 1:**
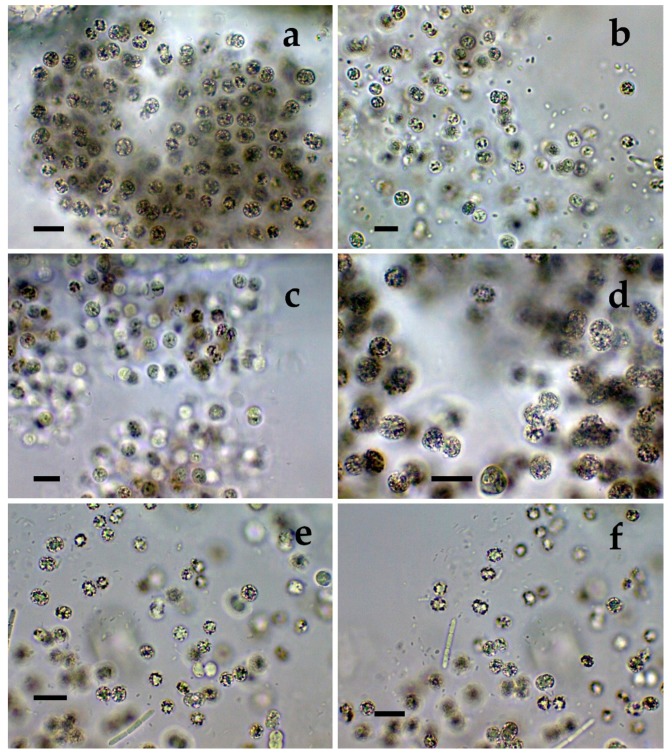
*Microcystis* from different Bulgarian waterbodies: (**a**,**b**) *M. aeruginosa* from Durankulak site 2; (**c**,**d**) *M. aeruginosa* from from Durankulak site 3; (**e**,**f**) *M. aeruginosa* from from Durankulak site 3 with filaments of *Pseudanabaena mucicola.* The scale bar equals 10 µm.

**Figure 2:**
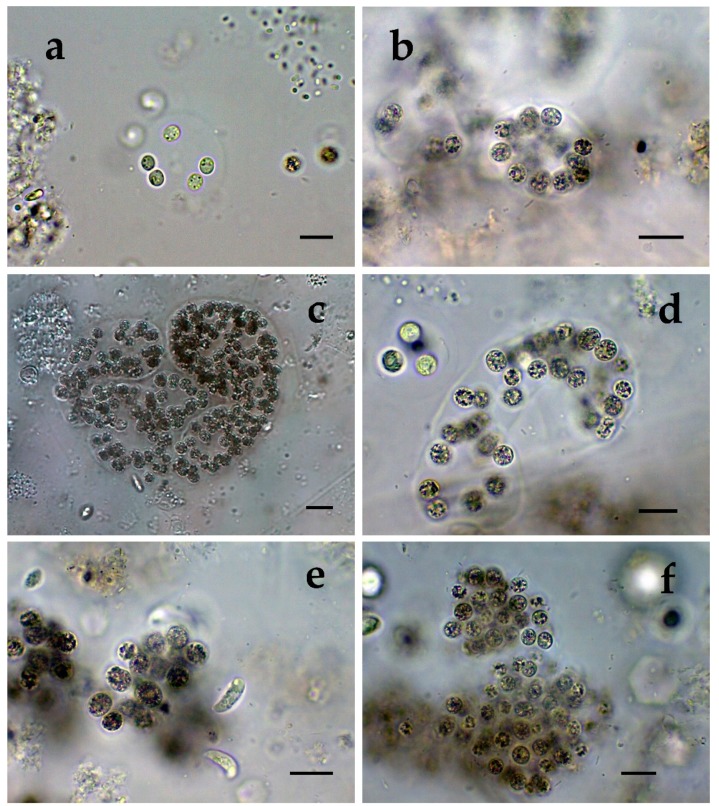
*Microcystis* from different Bulgarian waterbodies: (**a**) *M. wesenbergii* from Durankulak site 1; (**b**) *M. wesenbergii* from Durankulak site 2; (**c**) *M. wesenbergii* from Durankulak site 3; (**d**) *M. wesenbergii* from Durankulak site 4; (**e**,**f**) initial and developed colonies with transitional morphology between *M. wesenbergii* and *M. aeruginosa* in Durankulak site 4. The scale bar equals 10 µm.

**Figure 3:**
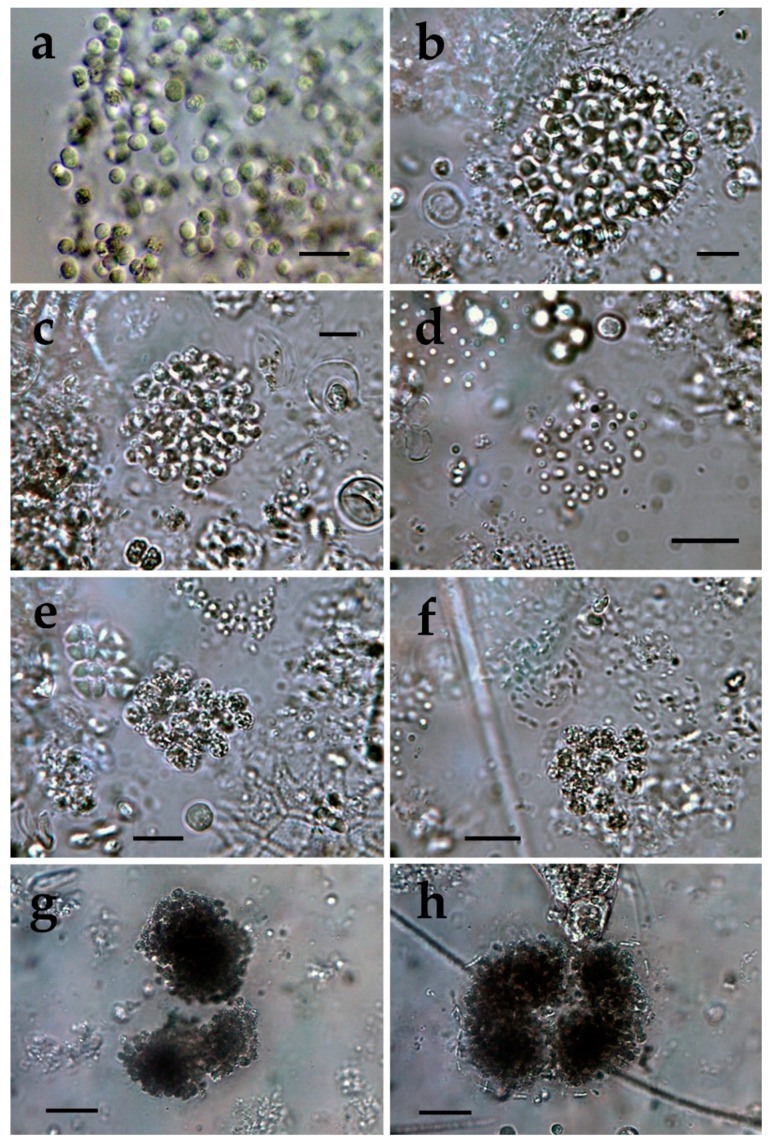
*Microcystis* from different Bulgarian waterbodies: (**a**) *M. smithii* in Durankulak site 3 (100×); (**b**) *M. botrys* in Durankulak site 2 (100×); (**c**) *M. flos-aquae* in Durankulak site 2 (100×); (**d**) *M. natans* in Durankulak site 2 (100×); (**e**,**f**) Initial Microcystis colonies with densely packed cells without visible mucilage in Durankulak site 2 (100×); (**g**) Undetermined *Microcystis* colonies with densely packed cells in Durankulak site 4 (40×); (**h**) Undetermined *Microcystis* colonies with densely packed cells with filaments of *Pseudanabaena mucicola* on the outer mucilage margin in Durankulak site 4 (40×). The scale bar equals 10 µm.

The richest diversity of *Microcystis* was found in the coastal lake Durankulak. The six species identified there represented 14% of the cyanoprokaryotes in the lake. Two of the species were common in all four sampled sites: *Microcystis aeruginosa* (Figure 1a–f) and *M. wesenbergii* (Figure 2a–f), but occurred in different amounts. *M. aeruginosa* was well represented and generally similar in its frequency, except in site 3, where it was much rarer. The morphological variability was significant, ranging from almost spherical colonies of more densely packed cells (Figure 1a) to irregular colonies with more spread cells (Figure 1b), with smaller (Figure 1c) or bigger cells (Figure 1d). Only in site 3, some colonies contained *Pseudanabaena mucicola* (Naumann & Huber-Pestalozzi) Schwabe (Figure 1e,f). *Microcystis wesenbergii* (Figure 2a–f) occurred in extremely low amounts in all of the sites. In site 1 also its separate cells were found, and in site 4 we observed initial and developed colonies with morphology transitional between *M. wesenbergii* and *M. aeruginosa*, which lack the typical outer margin (Figure 2e,f). Few colonies of *Microcystis smithii* were found only in site 3 (Figure 3a).

Typical *Microcystis botrys* was very rarely found only in the lake site 2 (Figure 3b), where we saw also single colonies of *M. flos-aquae* (Figure 3c) and *M. natans* (Figure 3d). In the same site 2 we found also some initial colonies with densely packed cells (almost black from numerous gas vesicles) without pronounced margin (Figure 3e,f). Similar but even more densely packed colonies with invisible margins were seen in the samples from site 4, some of which hosted filaments of *Pseudanabaena mucicola* (Figure 3g,h). The reliable identification of the colonies shown on Figure 3e–h, and of the separate cells was impossible by conventional LM.

**Figure 4:**
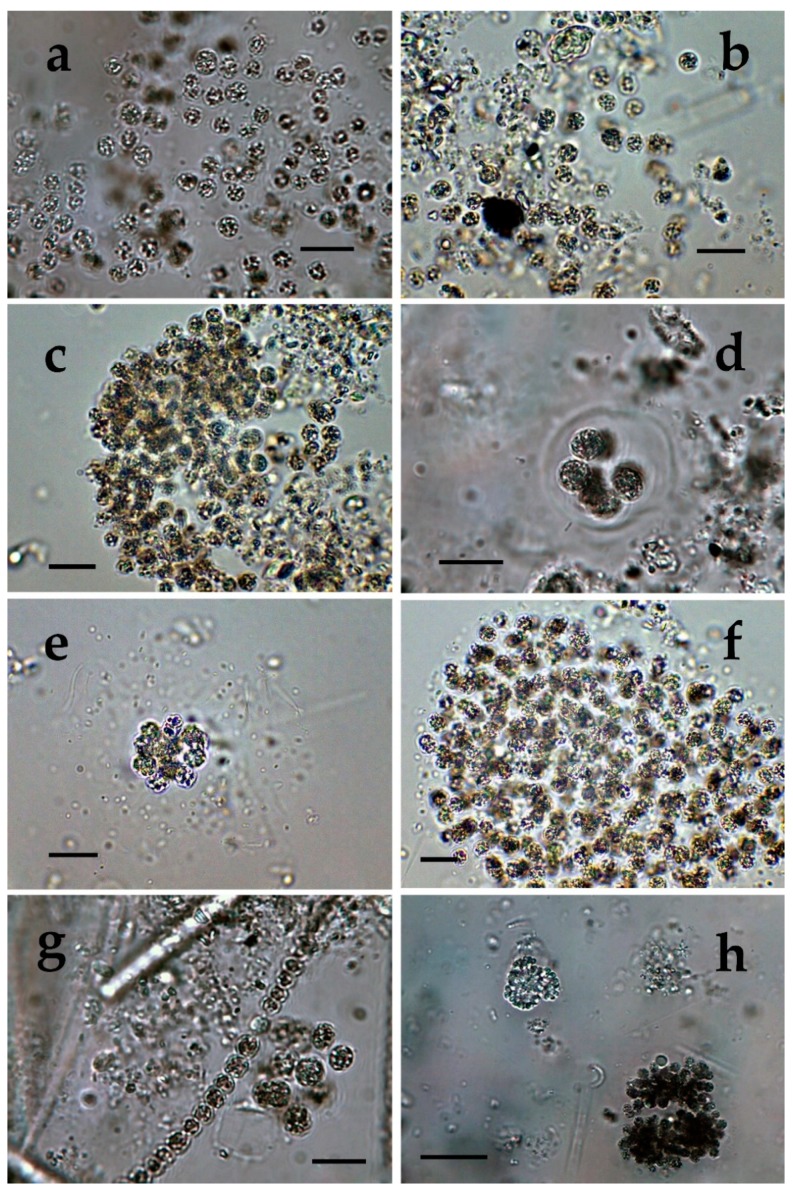
*Microcystis* from different Bulgarian waterbodies: (**a**) *M. aeruginosa* from Vaya site 1; (**b**,**c**) *M. aeruginosa* from Vaya 3; (**d**) Small colonies of *M. wesenbergii* from Vaya site 3; (**e**,**f**) Colonies with transitional morphology between *M. wesenbergii* and *M. aeruginosa* with fine mucilage instead of hard thick margin layer; (**g**) Initial colony with densely packed cells and without clearly visible mucilage in Vaya site 1 (100×); (**h**) Undetermined *Microcystis* colony with densely packed cells in Vaya site 3 (40×). The scale bar equals 10 µm.

**Figure 5:**
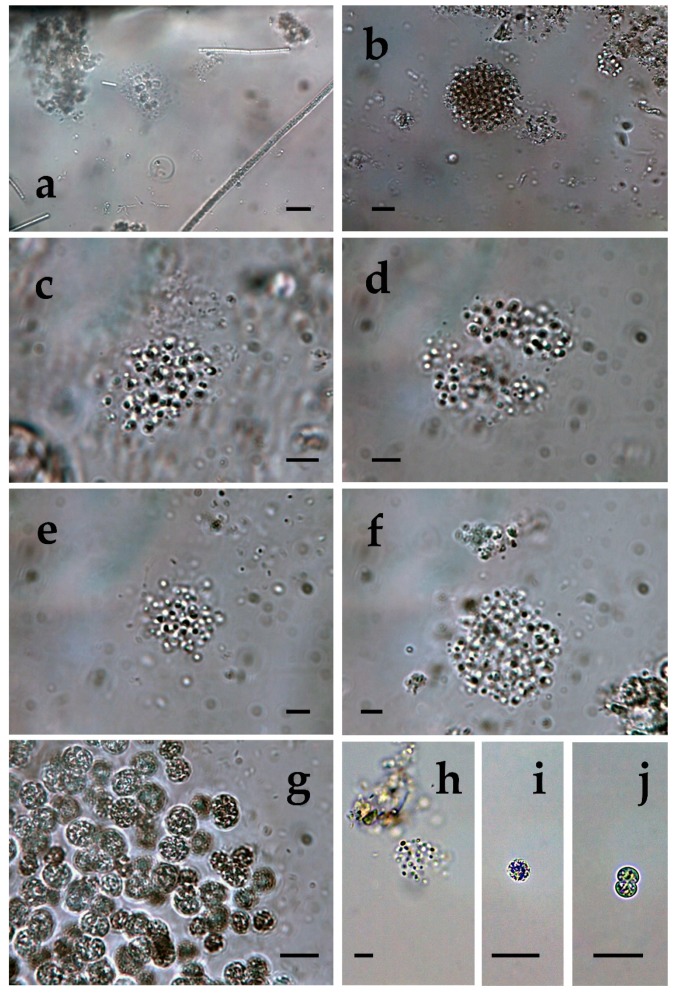
*Microcystis* from different Bulgarian waterbodies: (**a**) *M.* cf. *botrys* in Vaya site 2 (40×); (**b**) *M. flos-aquae* in Vaya site 3 (40×); (**c**,**d**) *M. novacekii* from Mandra site 1 (100×); (**e**,**f**) *M. novacekii* from Mandra site 3 (100×); (**g**) *M. aeruginosa* from Mandra site 3 (100×); (**h**) M. cf. *novacekii* from Poroy (100×); **i** – dividing cells from Poroy, which resemble *Synechocystis aquatilis* (100×); (**j**) Single separate cells of *Microcystis* from Poroy (100×). The scale bar equals 10 µm.

In the coastal Lake Vaya, we found four species of *Microcystis* (or, 9% of all lake cyanoprokaryotes). *M. aeruginosa* and *M. wesenbergii* were common for all three sampled sites. *M. aeruginosa* was relatively abundant in site 1 (Figure 4a) but was rare in lake sites 2 and 3. Its colonies in site 3 could be referred to two types—colonies with spread cells (Figure 4b) and colonies with more densely packed cells (Figure 4c). *M. wesenbergii* was rarely found in all three sites, where only small spherical colonies with few cells were seen (Figure 4d). In site 3, we observed young and developed colonies with morphology transitional between *M. wesenbergii* and *M. aeruginosa*, which were surrounded by fine mucilage and lack the typical thick outer margin (Figure 4e,f). In addition, some initial colonies without clearly visible mucilage, similar to the colonies in Durankulak site 2 (Figure 3e,f) were observed in Vaya sites 1 and 3 (Figure 4g), and undetermined colonies similar to those from Durankulak site 4 (Figure 3g) were observed in Vaya site 3 (Figure 4h), sometimes with spread nearby separate cells of the same type. In sites 1 and 2, a few small colonies with structured mucilage were tentatively identified as *M. botrys* (Figure 5a), and in site 3 a single colony was referred to *M. flos-aquae* (Figure 5b). Some small colonies similar to initial *M. novacekii* were seen in the samples from Vaya site 2, and singular dividing cells, resembling *Synechocystis aquatilis* Sauvageau, were seen in Vaya site 1.

In the coastal reservoir Mandra, colonies identified as *M. novacekii* (Figure 5c–f) were seen in sites 1 and 3, while *M. aeruginosa* was rarely found only in site 3 (Figure 5g). There its cells were with the largest dimensions (7–7.5 µm) in comparison with the colonies from all other WBs, where cell diameters ranged from 3.5 to 5 (6) µm. In the samples from site 2 we did not see any colonies or cells of *Microcystis.* Both *Microcystis* species represent 22% of cyanoprokaryote taxa in the reservoir.

In the coastal reservoir Poroy by LM we found small initial colonies, which resembled *Microcystis novacekii*, but shared features with the genera *Coelosphaerium* and *Coelomoron* (e.g., small spherical cells distributed on the outer colony layer)—Figure 5h. In the same samples, we saw some separate spherical cells with gas vesicles which could be related with both *Microcystis aeruginosa* and *M. wesenbergii* due to the overlapping dimensions of 5–6 µm (Figure 5j). In addition, there were some singular dividing cells, which could belong to the same two species but also strongly resembled *Synechocystis aquatilis* (Figure 5j). According to these results, we could suppose presence of two species, which represent 25% of the cyanoprokaryote diversity in this reservoir.

**Figure 6:**
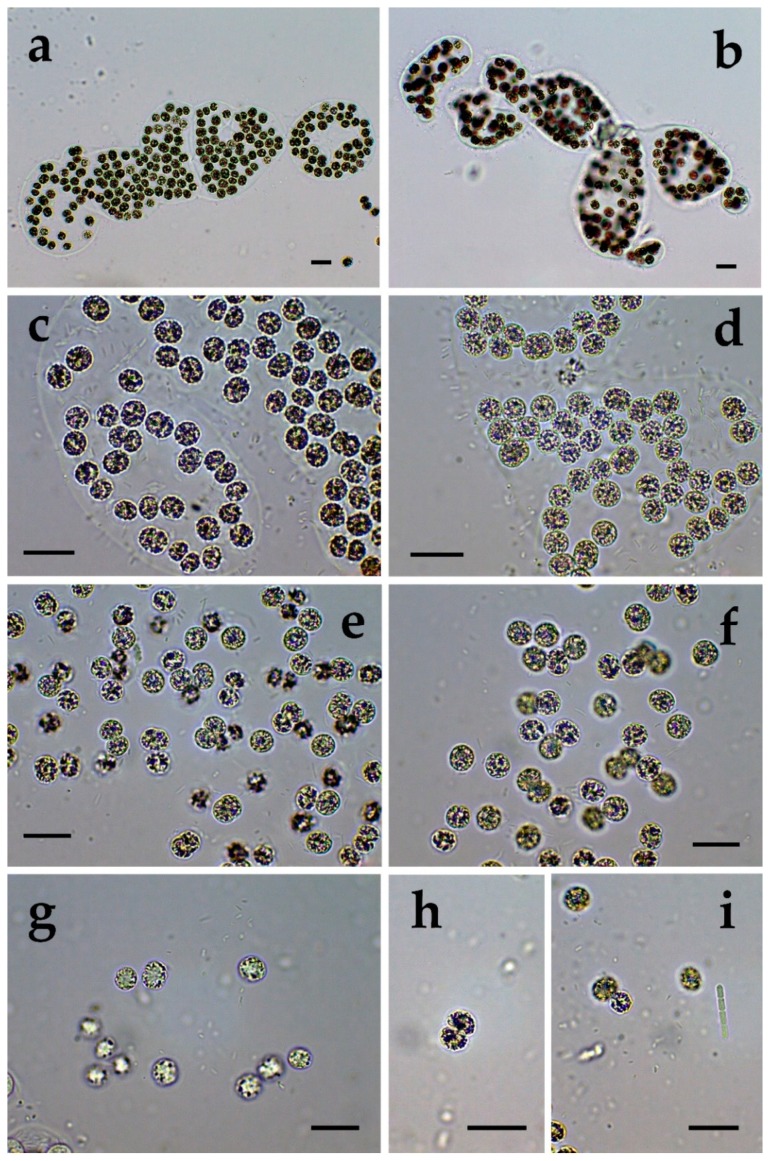
*Microcystis wesenbergii* from Sinyata Reka: (**a**,**b**) typical colonies (40×), (**c**,**d**) typical colonies (100×); (**e**–**i**) disintegrated colonies to separate cells (100×), where some of the separate dividing cells resemble *Synechocystis aquatilis* (**g**) and some groups of cells contain filaments of *Pseudanabaena mucicola* in the mucilage remnants (**i**). The scale bar equals 10 µm.

**Figure 7:**
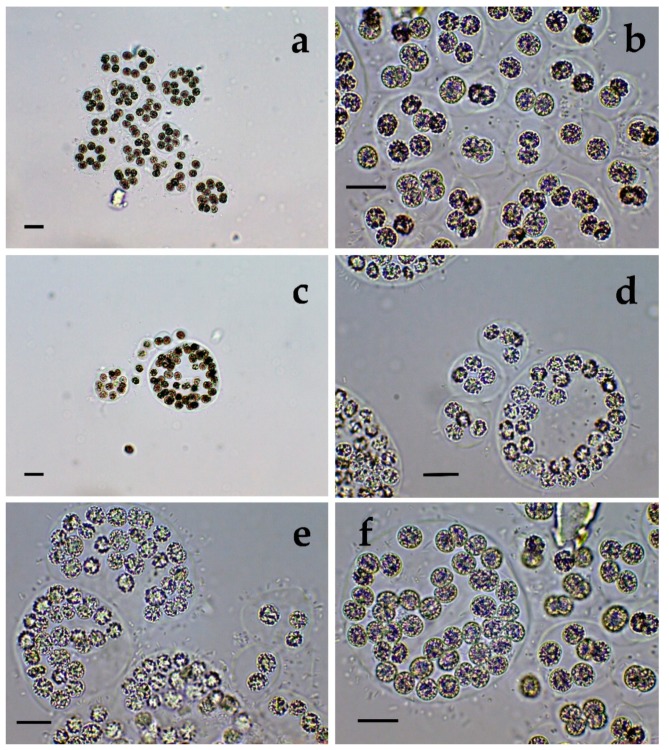
*Microcystis wesenbergii* from Sinyata Reka: Young spherical colonies (**a**) 40×, (**b**) 100×; (**c**) different colonies and separate cell (40×); Different colonies at 100× (**d**) without epigloeoic bacteria; (**e**) with epigloeic bacteria; (**f**) Transitions during colonies disintegration to small colonies and different stages from cells, surrounded by outer hard mucilage layer to completely separate cells without mucilage (100×). The scale bar equals 10 µm.

**Figure 8:**
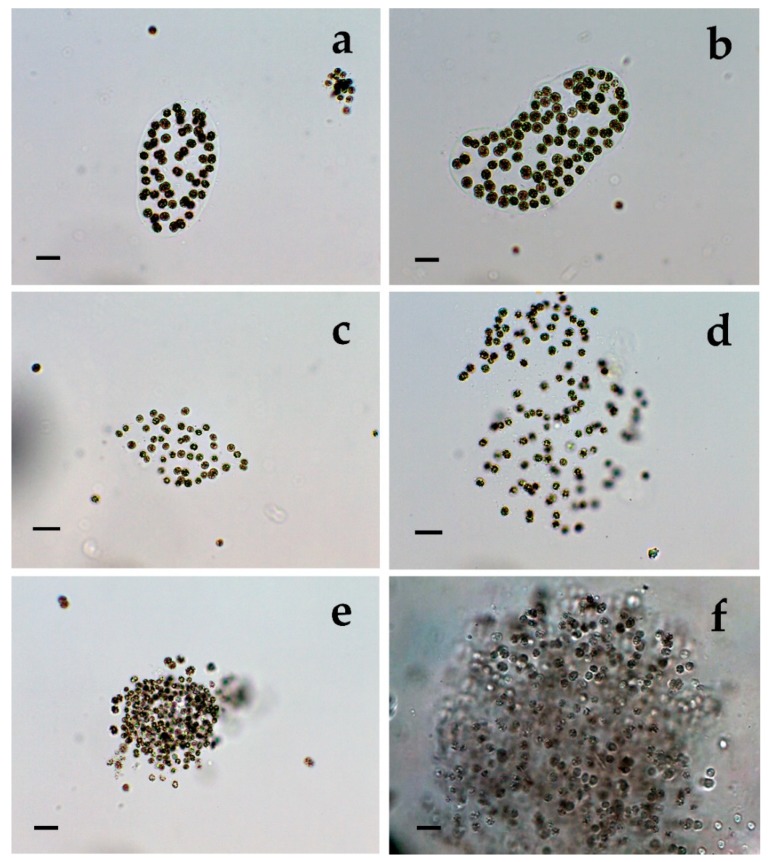
*Microcystis wesenbergii* from Sinyata Reka: (**a**,**b**) typical colonies and separate cells (40×), (**c**,**d**) irregular colonies with invisible margin, transitional between *M. wesenbergii* and *M. aeruginosa*, and separate cells (40×); (**e**,**f**) spherical colonies which shared morphological features with *Microcystis flos-aquae* and *M*. *novacekii* (40×). The scale bar equals 10 µm.

In the both sites of the small inland reservoir Sinyata Reka, we found almost a clear culture of well-pronounced, typical perforated and irregular colonies of Microcystis wesenbergii accompanied with many young spherical colonies and some separate cells from disintegrated colonies (with all transitions from rarely visible remnants of the hard colonial mucilage margin to completely free from mucilage single cells (Figure 6a–i, Figure 7a–f and Figure 8a–d). Some of the single dividing cells, when found separately, resembled *Synechocystis aquatilis* and could be easily misidentified (Figure 6g). In addition, we observed a few colonies without defined margin of two different types: (1) colonies which slightly resembled the irregular colonies of *Microcystis aeruginosa*, but were without holes (Figure 8c,d); (2) spherical colonies which shared morphological features with *Microcystis flos-aquae* and *M. novacekii* (Figure 8e,f). There were also clearly visible differences due to presence or absence of epiphytic/epigloeic bacteria on the mucilage margin (Figure 6c,d), and extremely rarely *Pseudanabaena mucicola* was associated with the disintegrated colonial remnants (Figure 6i).

### 2.2. Results from PCR Analysis for Microcystin and Nodularin-Producing Strains

**Figure 9:**
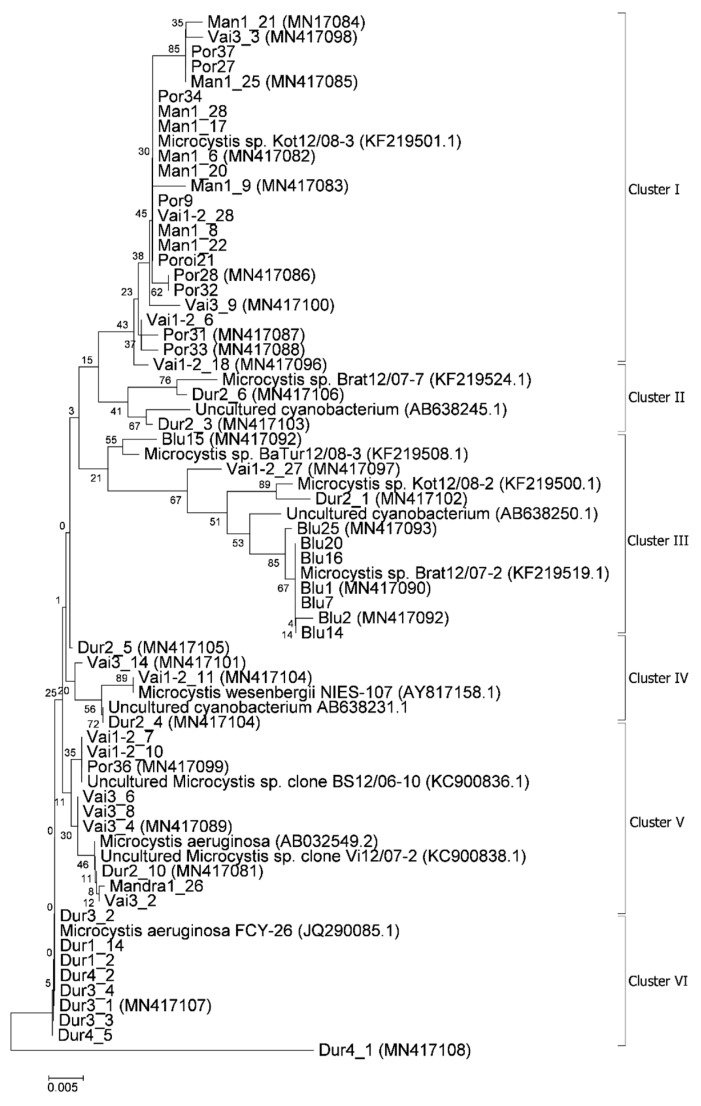
Neighbor-joining phylogenetic tree constructed using nucleotides sequences from nine library samples and closest sequences retrieved after Blast search in NCBI database with indication of their accession number in NCBI. Bootstrap values are shown at branch points (percentage of 1050 resamplings). Legend: Man—Reservoir Mandra; Dur—Lake Durankulak; Vai—Lake Vaya; Por—Reservoir Poroy; Blu—Reservoir Sinyata Reka (=Blue River). For the identical sequences (IS), obtained during this study, only one accession number received from NCBI is provided in each cluster or sub-cluster. The IS from the sites Mandra 1, Poroy, and Vaya 1–2 (cluster I) are with accession number MN417082, the IS from the sites Durankulak 1, 3, and 4 (cluster VI) are with accession number MN417107, the IS from Poroy and Vaya 1–2 (cluster V) are with accession number MN417089, and the IS from Mandra 1 and Poroy (cluster I) are with accession number MN417085.

The HEPF × HEPR synthetase-gene-specific pair of primers was used to identify MC- and NOD-producing genotypes in the phytoplankton samples from 17 studied sites of nine Bulgarian WBs. The amplification of the expected 472 bp fragment was found in the samples from nine sites of Sinyata Reka, Poroy, Vaya, Mandra, and Durankulak (Figure 9). After the isolation of DNA fragments from these positive samples and their cloning into plasmid vector nine *mcyDNA*-clone libraries were constructed. The sequence analysis of the totally obtained 57 *mcyDNA* clones resulted in identification of 28 sequences homologous with the *mcyE* module (Figure 9). NOD sequences were not detected.

According to the BLAST search [39], 31 (54%) from the 57 obtained sequences had 100% identity with the Genbank (abbreviated hereafter as NCBI) [40] sequences of known strains and could be affiliated to them. Other 25 (44%) showed high level homology (99% identity), and only one strain (namely, Dur 4_1—Figure 9) was more distant, showing lower identity (96%) to the known *mcyE* sequences. All the analyzed 57 *mcyE* sequences and their corresponding higher homology GenBank sequences were used in the phylogenetic assay. In the constructed phylogenetic tree six main clusters were formed (Figure 9). The combined results from GenBank search and phylogenetic analysis demonstrated that from the all 57 obtained sequences, 56 clearly belonged to the genus *Microcystis,* while for now, the affiliation of the single distant low homologous (96%) sequence Dur4_1 was impossible. Most of the 56 sequences (44, or 79%) were affiliated to uncultured or unidentified to species level strains of the genus *Microcystis* and only 12 of them (21%) could be referred to the strains of two distinct species—*M. aeruginosa* and *M. wesenbergii* (Figure 9).

The phylogenetic tree, constructed from the analyzed *mcyE* sequences and their homologous representatives from NCBI, demonstrated the complexity of the isolated sequences pool (Figure 9). This complexity reflects the relevant rich biodiversity of *Microcystis* strains in the studied WBs. It ranged between the highest level of 14 sequences obtained from the sites of the coastal lake Vaya and spread in four different clusters (I, III, IV, and V), and the lowest biodiversity in the inland reservoirs Sinyata Reka (with all eight sequences concentrated in cluster III). Rich diversity was found to occur also in the sequences from the coastal lake Durankulak (13), most of which were obtained from sites 1,3, and 4 and were included into the single cluster VI, while the sequences from site 2 were spread in three clusters (II, IV, and V). Nine sequences from the coastal reservoirs Poroy and Mandra were concentrated in one cluster (I) and only one sequence from both habitats was incorporated in cluster V (Figure 9). As it was mentioned above, the sequence with the 96% homology from site Durankulak 4, included separately in the phylogenetic tree, at present could not be assigned to any known genus.

## 3. Discussion

The taxonomic results obtained by LM during this study corroborate the HPLC data on the phytoplankton composition of the studied WBs and chemically detected cyanotoxins [4]. The results from conducted molecular-genetic analysis are also in general accordance with data on cyanotoxins, except the lack of MCs over the detection limit of the methods used in Poroy, Vaya, and Mandra [4], and recent discovery of toxigenic *mcy* sequences in these WBs. The finding of toxigenic sequences in the samples where MCs were not detected by standard methods is not unusual and can be explained with the quite low *Microcystis* amounts found there by LM, with the temporal character of *mcy* gene expression patterns (e.g., [41]) and other factors that condition the toxin production, including the growth phase of the populations [42]. The lack of NODs in the checked WBs [4] is also in accordance with the negative PCR signal for NOD-producing genes obtained in this study and the absence of its causative agents, *Nodularia* species [22], in the phytoplankton samples processed by LM. We note these results also considering the previous broad distribution of different species of *Nodularia* in coastal Bulgarian waterbodies and the former mass development of *N. spumigena* in Vaya (for details see [3]).

*Microcystis* represents one of the most proliferative bloom-forming genera, reported from more than 108 countries and on all continents [38,43,44]. From the 11 *Microcystis* morphospecies, distinguishable by LM and accepted as distributed in Europe [36,37], we found 7 in the studied WBs (Table 1, Figure 1, Figure 2, Figure 3, Figure 4, Figure 5, Figure 6, Figure 7 and Figure 8). Out of those seven WBs, three *Microcystis* morphospecies—namely *Microcystis natans*, *M. smithii*, and *M. botrys*—are more common in northern parts of Europe and in large clear lakes, while *M. aeruginosa*, *M. flos-aquae*, *M. novacekii*, and *M. wesenbergii* are more widely distributed and common in mesotrophic and eutrophic WBs, where they often form water blooms or participate in them [36]. In our study, the first three species (*M. natans*, *M. smithii*, and *M. botrys*) were rarely found and always in low abundance, which allows tentatively to suggest their alien character for the investigated WBs and Bulgaria. *M. natans* and *M. botrys* were rarely found in the country, the first in the reservoir Pchelina and in the lake Vaya [45], and the second in the reservoir Mandra [46], but this is the first report of *M. smithii* for Bulgaria.

Generally, both LM and molecular genetic approaches demonstrated presence of *Microcystis* in five of the WBs and confirmed the uneven distribution of its clones and toxigenic representatives in the studied sites. The species of the genus were not found by both LM and PCR-based methods in four of the WBs—namely lakes Uzungeren, Shabla, Ezerets, and in the reservoir Aheloy, as well as in the site 2 of the reservoir Mandra. In the other five studied WBs, 56 clones of *Microcystis* were identified as spread in six clusters according to their homology with *mcyE*-based sequences (Figure 9). The fact that only 12 (21%) of the sequences were affiliated to the strains of distinct species (*M. aeruginosa* and *M. wesenbergii*) provided a great challenge to assign all obtained toxigenic genotypes to certain *Microcystis* taxa. On the one hand, the low number of affiliations was due to the recent general low availability of genomic sequences from cyanobacteria when compared to other prokaryotes [47]. In the same time, five of the NCBI sequences used in the obtained phylogenetic tree (Figure 9) were based on metagenomic data (genetic material recovered directly from uncultured organisms from environmental samples—[48]). Despite the increased application of these data in modern studies based on the common difficulties, or even impossibility, to culture some cyanobacteria [47], their use in identification work is problematic because of the lack of morphological descriptions for most of the sequenced uncultured strains. The same is the case of most axenic strains with fixed sequences, registered in the NCBI and used for obtaining the phylogenetic tree. Another important factor to note is the general problem of lost value of some phylogenetic constructions caused by using sequences from strains which have not passed taxonomic revision and yet have incorrect or arbitrary names [10]. Despite considering all these possible problems and needed caution in interpretations, at present, according to the comparison of our results obtained by both methods, it is possible to suppose that:

(1) Cluster I contains mainly strains identical to *Microcystis* sp. Kot12/08-3 (NCBI:txid1402958), which are similar in LM to *Microcystis novacekii,* from which the best morphologically expressed features of the colonies were found in the reservoir Mandra (Figure 5c–f);
(2) Because of the finding of *Microcystis natans* only in Durankulak site 2 (Figure 3d) cluster II most probably comprises its two strains which are close to *Microcystis* sp. Brat 12/07-7 (NCBI:txid1402954) and uncultured cyanobacterium (AB638245.1);
(3) Cluster III contained a group of seven strains of typical *Microcystis wesenbergii* (identical with *Microcystis* sp. Brat12/07-2, NCBI:txid1402949), some of which are capable of easy dissolving to separate cells accompanied with some morphological transitions to *Microcystis aeruginosa* during the disintegration of the colonies (Figure 4e,f) and four other groups of strains with disputable from genetic point of view affiliation despite the fact that by LM similar strains of *M. wesenbergii* were seen in the reservoir Sinyata Reka and in the lakes Durankulak (site 2) and Vaya (sites 1 and 2);
(4) Cluster IV contained two groups of strains: (a) a strain of *Microcystis wesenbergii* (100% identical to *Microcystis wesenbergii* NIES-107, NCBI:txid315483) with sharp, hard and well-pronounced margin of the colonies, which easily defragment to small spherical initial colonies in the lake Vaya (Figure 4d); (b) strains from Vaya site 3 and Durankulak site 2 (the last similar to Uncultured cyanobacterium AB638231), for which it is possible to suppose close affiliation to *Microcystis aeruginosa* from cluster VI according to their genetic distances but which could not be clearly separated by LM;
(5) Cluster V comprises generally of morphologically different strains of *Microcystis aeruginosa* with a sub-cluster of clones distributed in Vaya 1–2 and Poroy, the colonies of which are easily disintegrating in separate cells, often dividing in twos, which strongly resemble *Synechocystis aquatilis* and are impossible to be reliably ascertained to *M. wesenbergii* or *M. aeruginosa* but obviously are genetically close to uncultured *Microcystis* sp. clone BS12/06-10; another close sub-cluster is formed by strains found in Mandra 1, Durankulak 2, and Vaya 3 and identical with to *Microcystis aeruginosa* (AB032549.2) and uncultured *Microcystis* sp. clone Vi12/07-2, which could not be clearly separated by LM;
(6) Cluster VI contains the most typical but strongly variable *Microcystis aeruginosa* strains, which were found mainly in the lake Durankulak (Figure 1a–f) and were identical to *Microcystis aeruginosa* FCY-26 (NCBI:txid1150859).

At present, we cannot assign to any cluster the morphologically identified *Microcystis smithii*, found only in site 3 of the lake Durankulak (Figure 3a; Table 1), and cannot link the last separate sequence from Durankulak site 4 (Dur4_1 on Figure 9) to a certain morphological strain. The most clearly morphologically different colonies found in Durankulak site 4 (Figure 3g,h) had similarities with some colonies found in Vaya site 3 (Figure 4h) but their sequences did not group in any common cluster on Figure 9. The same lack of common phylogenetic grouping could be outlined for *Microcystis botrys,* which was found in Durankulak site 2 and Vaya sites 1 and 2 (Figure 3b and Figure 5a), as well as for *M. flos-aquae* which was found in Durankulak site 2 and Vaya site 3 (Figure 3c and Figure 5b).

The distribution of sequences in the obtained phylogenetic tree did not match completely with their geographic distribution in coastal and inland parts of the country, and this result is in accordance with the earlier demonstrated lack of clear biogeographical pattern in the variability of sequenced *Microcystis aeruginosa* genomes [49]. The different spread of *Microcystis* morphospecies in the studied WBs (Table 1) corroborates previous data on the distribution of the cyanoprokaryotes in the country [2,3].

Despite the general accordance in all the results, we have to note that there was not complete coincidence of data obtained by both applied methods since we found seven morphospecies of *Microcystis*, but six clusters based on PCR. Moreover, in one case (namely Mandra site 3) by LM we clearly identified the presence of two *Microcystis* species (*M*. *aeruginosa* and *M. novacekii—*Table 1) but the samples from the same site gave a negative PCR-signal. PCR data also did not indicate presence of *M. wesenbergii* in the site Durankulak 4, where it was found in typical colonies with hard outer mucilage margin and therefore inevitably determined by LM (Figure 2d). The same were the mentioned above cases of rare findings by LM of *M. botrys* and *M. flos-aquae,* which we could not assign to any cluster of the phylogenetic tree. The explanation for these discrepancies could be found in the extremely low quantities of the *Microcysti*s colonies in the relevant sites observed by LM and in the eventual presence of non-toxic strains. It is possible to suggest the same reasons for not obtaining a clear PCR-signal for *M. smithii,* which was identified by LM in Durankulak site 3 (Figure 3a). Considering this result, it is well to remember that 16S rRNA gene sequences of five strains of *M. smithii* isolated from Lake Dishui, China, intermixed with strains of other morphospecies [50]. Finally, we have to note that the reliable LM identification of morphologically typical *Microcystis wesenbergii* in the reservoir Sinyata Reka (Figure 9) was not inevitably confirmed by PCR-data because of the lacking morphological description of *Microcystis* sp. Brat 12/07-7 (NCBI:txid1402949).

All the results obtained in this study support the idea of rich intrageneric diversity with many transitions in the colonial morphology in the genus *Microcystis.* This is in accordance with the earlier accent on the taxonomic problems in LM identification of *Microcysti*s caused by the variability of colonies with overlapping of the limits usually accepted for a particular morphospecies [37,38,51], as well as with the recent hypothesis that all *Microcystis* morphospecies are may be different morphotypes of just one genetically consistent species and their phenotypic plasticity is caused by environmental variables [8]. Considering also the earlier works on *M. aaeruginosa, M. smithii, M. novacekii,* and *M. wesenbergii,* based on 16S rRNA, or 16S rRNA–23S rRNA ITS and cpcBA-IGB regions which suggested monophyletic identity, or demonstrated lack of differences among morphospecies or showed their intermixed phylogenetic positions [50,52,53,54], we support the opinion that taxonomic revision of *Microcystis* is needed.

When discussing the differences in the results obtained by different methods, we have to underline also that *mcyE* sequences proved the presence of MC-producing *Microcystis* strains in Poroy, Vaya, and Mandra, where MCs were not detected by conventional methods [4] but *Microcystis* colonies were identified by LM. Once more this result shows the efficiency of the PCR-based method in cases of low abundance of toxigenic strains. Therefore, we can underline the sensitivity of molecular methods for identifying of MCs, but in the same time, based on all our results, we support the broadly shared opinion that until a single unique method is adopted, a combination of different approaches is more desirable, or even necessary in studies of CyanoHABs.

The current applied approach that combines two different methods for the study of cyanobacterial blooms has the benefit of relating the presence of the toxigenic *mcyE* genes to the different morphospecies confirmed by LM. Toxic strains are world-wide known for *M. aeruginosa* and have been found in *M. botrys, M. flos-aquae*, and *M. novacekii* [55]*,* while according to our knowledge they have not been reported for *M. natans,* and data on *M. wesenbergii* remain disputable (e.g., [2,36,38,56,57,58]). In Bulgaria, *M. wesenbergii* occurred among the most spread cyanoprokaryotes, and similarly to *M. aeruginosa*, *M. flos-aquae*, and *M. natans* it was found in the samples containing toxins [2]. Moreover, *M. wesenbergii* was the most often recorded species in the toxic samples found in the country during the analyzed 15-year period [2]. The results from the genetic analysis carried in this study together with our LM data, allowed strongly to suppose the relation of the toxic strains in cluster III (Figure 9) with the nuisance bloom with MCs detected in the reservoir Sinyata Reka [4] and formed by morphologically identified dominant *M. wesenbergii* (Figure 6, Figure 7 and Figure 8), different from the only published with a sequence *M. wesenbergii* strain NIES-107. The results from cluster IV proved the presence of another toxic *M. wesenbergii* strain in Vaya sites 1 and 2, which is similar to the abovementioned strain NIES-107. In addition, data obtained in this study allow us to suppose the existence of toxic strains of *M. natans,* which in our opinion is represented in cluster II. This suggestion finds support in a previous publication on Bulgarian WBs, according to which *M. natans* was found among the algal dominants during three CyanoHABs with MCs in the lake Vaya and in the reservoirs Pchelina and Bistritsa [45]. As it could be seen from Figure 9, and as it could be expected from all earlier data on *Microcystis* toxicity cited above [55], most of the *M. aeruginosa* strains found during the study were toxic. However, we did not find evidence for presence of toxic strains containing *mcyE* genes of *Microcystis botrys, M. flos-aquae*, and *M. smithii* strains and for now were not able to resolve their identification based on the used HEPF x HEPR pair of primers.

## 4. Conclusions

The results obtained from application of the polyphasic approach in this particular study allowed us to state the presence of toxic *Microcystis* strains in five from the nine studied shallow WBs of Bulgaria, in some of which both blooms and MCs have been confirmed by other methods [4]. Moreover, using the polyphasic approach, we confirmed the well-known presence of toxic *M. aeruginosa* strains, proved the presence of toxigenic strains in *M. wesenbergii* and supposed their existence in *M. natans*. The general accordance in the results allows to confirm the idea that genetic sequencing, and the HEPF × HEPR pair of primers in particular, can efficiently serve in water monitoring, employing a cultivation-independent approach even in cases of low abundance of toxigenic strains. We demonstrated also that until a single unique method is adopted, a combination of different approaches is more desirable, or even necessary in studies of CyanoHABs. However, the affiliation of most of the isolated *mycE* strains to genus level due to unclassified species sequences, suggested that the currently available cyanobacterial genomic sequence data are still insufficient to resolve fully the species phylogenetic identification of the isolated *mcy* pool sequences with covering the whole range of *mcyE* diversity and toxicity. Our results also strongly support the need for taxonomic revision of the genus *Microcystis.*

## 5. Materials and Methods

### 5.1. Sites and Sampling

**Figure 10:**
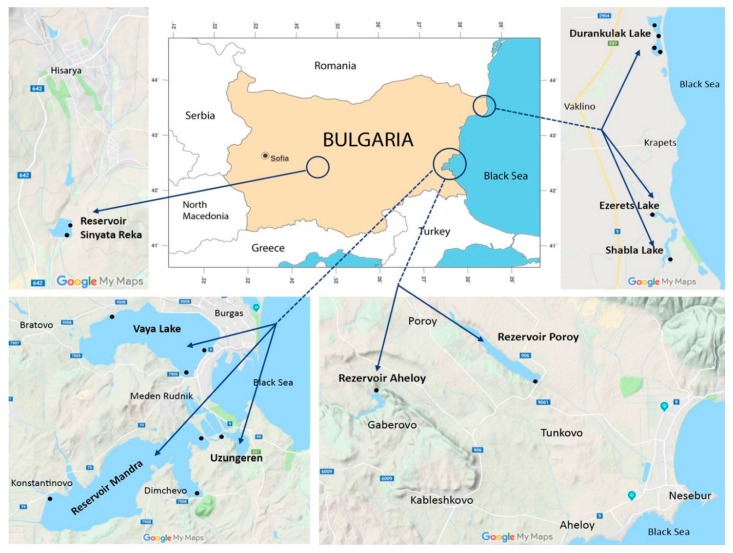
Map of Bulgaria showing the sampling sites (modified after http://www.ginkgomaps.com and Google Maps, accessed 6 November 2019).

The study was carried out in a single campaign from 21–25 June 2018 in nine shallow WBs situated in Central and Eastern Bulgaria (Figure 10, Table 2). Detailed descriptions on the morphometry, hydrology, historical development, usage, conservational status, and biodiversity of each of the WBs are provided in the Inventory of Bulgarian wetlands and their biodiversity [31]. Therefore, in Table 2 this unique inventory number of each WB from this database (IBWXXXX) is provided.

The sampling was preceded by sending a drone (DJI Mavic Pro, Shenzhen, Guangdong, China, Model: M1P GL200A) supplied with a photo camera to observe and document the whole WB and eventual hot spots with visible differences in the color as indicators of cyanoblooms. The spots/areas of different color were chosen for sampling or, in case of visible water homogeneity, the sites from our previous studies were repeated for each WB (for details see [4]). Therefore, the number of sampling sites (provided in Table 2) in each water body varied from 1 to 4. All the chosen 17 sites were reached by inflatable boats with one or two places, with motor and oars, used according to the site circumstances. The site coordinates, altitude, water temperature, pH, water hardness (TDS), oxygen content (DO), and conductivity were measured in situ by Aquameter AM-200 and Aquaprobe AP-2000 from Aquaread water monitoring instruments, 2012 Aquaread Ltd (Broadstairs, UK). Total nitrogen (TN) and phosphorus (TP) were measured ex situ using Aqualytic AL410 Photometer from AQUALYTIC^®^, Dortmund, Germany. The water transparency was measured using Secchi disk. All results, together with detailed data on cyanotoxins found were published [4].

Phytoplankton samples for taxonomic identification and for molecular-genetic studies (each in a volume of 0.5 L) were collected from the water surface (0–20 cm). The phytoplankton samples were fixed immediately with 2% formalin and thus transported to the lab, where they were further proceeded by sedimentation method. The samples for PCR-studies were filtered in some hours after collection and the obtained filters were transported to the lab in sterile plastic tubes in a box with dry ice.

### 5.2. Phytoplankton Identification by Conventional Light Microscopy (LM)

In the lab, the microscopic work was done mainly under magnification 100× and immersion on 52 non-permanent slides on Motic BA 4000 microscope with camera Moticam 2000, and later on 22 non-permanent slides on Motic B1 microscope with camera Moticam 2.0 mp. Both cameras were supplied by Motic Images 2 Plus software program.

Taxonomic identification of cyanoprokaryotes followed the standard European taxonomic literature [32,33,34,35,36,59] with updates from AlgaeBase [60], CyanoDB [61] and relevant modern taxonomic papers. Traditional morphological features for distinguishing *Microcystis* ‘species’ include the colony form, mucilage structure, cell diameter, density and organization of cells within the colony, facultative or obligatory occurrence of aerotopes, and the term ‘morphospecies’ has become widely used for species recognized on such morphological criteria (e.g., [32,33,34,35,36,37,38]).

Quantitative contribution of the different species to the biomass was estimated using the Thoma-counting chamber and method of the stereometrical approximations [2,62].

### 5.3. Molecular Studies

The molecular study was conducted by sequence analysis of PCR amplified fragments of toxic microcystin synthase gene *mcyE*. As already mentioned, this targeted gene belong to the gene clusters involved in the biosynthesis of MCs (*mcyA*-*E*), which are widely used due to their usefulness for the early detection of potentially toxic cells even when the toxin concentrations are too low to be detected [21]. Due to lack of universal primers (e.g., [26,63]), for this study, the *mcyE* gene was chosen out of the other potentially usable *mcy* genes because of its reliable biomarker character for detection of MC-producing cyanoprokaryotes and its strong indicator properties for identifying of potential risk from MCs, even in water bodies comprising mixed assemblages of toxic and non-toxic cyanobacteria (e.g., [64,65,66,67,68,69]). Moreover, the similarity between MCs and NODs biosynthesis pathways [22,23] enabled the development of molecular detection methods for identifying all the main producers of MCs and NODs in environmental samples based on *mcyE*-gene and the orthologous nodularin synthetase gene F (*ndaF*) sequences with specific detection of the *mcyE/ndaF* gene pairs [65,70,71]. The application of primers specific to *mcyE* genes showed advantages in toxigenicity typing [72] especially after the amplified aminotransferase (AMT) domain of *mcyE* using HEP primers revealed that PCR amplification and hepatotoxin production was 100% correlated [65].

A few hours after collection, the samples for molecular-genetic analysis were filtered true 45 µm cellulose filters Whatman NC45 ST/Sterile EO. In the lab, the total DNA was isolated from the filters following the protocol of Sigma Genomic DNA Purification Mini Kit (Sigma).

DNA was amplified using primer combination HEPF (5′TTTGGGGTTAACTTTTTTGGGCATAGTC-3′) × HEPR (5′AATTCTTGAGGCTGTAAATCGGGTTT-3′) [65]. The PCR amplification was performed in a 25 µL volume containing 10 pmol primers; 0.16 mM dNTP’s; 1.25 units Taq polymerase, 0.75 mM MgCI_2_ and 10× PCR buffer supplied by FastStart High Fidelity PCR System (Roche, Basel, Switzerland). The amplification of DNA was done in a thermal cycler QB-96 (Qianta Biotech, Byfleet, Surrey, UK) under the following PCR conditions: denaturation at 95 °C for 5 min, 35 cycles of denaturation (30 s at 95 °C), annealing at 57 °C for 30 s, extension at 72 °C for 40 s, and a final extension at 72 °C for 5 min. The resulting PCR products were purified using Sigma Gel GenElute Gel Extraction Kit (Sigma, St. Louis, MO, USA), following the manufacturer’s instructions.

The amplification products obtained were subsequently cloned using with CloneJET PCR Cloning Kit (Thermo Scientific, Waltham, MA, USA). Recombinant plasmids were isolated using Sigma Plasmid Miniprep Kit. For each site or WB between 6 and 10 clones were selected and sequenced by Macrogen Inc (Seol, Korea). The obtained sequences were processed with Vector NTI 11.5 software and used for BLAST search [39] in the NCBI data base [40]. All the 28 sequences obtained during this study were submitted to NCBI [40] and the accession numbers from MN417081 to MN417108 were received. The phylogenetic tree was constructed by Neighbor-joining method applying Mega 6.06 software [73]. In the resultant tree, the accession numbers of the strains are shown in brackets, while in the text the taxonomic identification number in NCBI is indicated in accordance with the requirements of NCBI [40].

## Figures and Tables

**Table 1:** Distribution of *Microcystis* taxa in the studied Bulgarian waterbodies (WBs).

WBN and IBW	SAN	MA	MB	MF	MNs	MNv	MS	MW	SS	SL	TCs
Reservoir Sinyata Reka—SR (IBW1890)	SR1							d	x		x
	SR2							d	x		x
Vaya Lake—VA (IBW0191)	VA1	x	r					x		x	x
	VA2	x	r			r		x	x		x
	VA3	x		r				x			
Reservoir Mandra—MN (IBW1720)	MN1					x					
	MN3	x				x					
Reservoir Poroy (IBW3038)						x			x	r	
Durankulak Lake—DR (IBW0216)	DR1	x						x	x		x
	DR2	x	r	r	r			r			x
	DR3	x					r	r			x
	DR4	x						r			x

WBN—name of the WB; IBW—number of the WB in the Inventory of Bulgarian Wetlands [31]; SAN—site abbreviation and number; MA—*Microcystis aeruginosa*, MB—*Microcystis botrys*, MF—*Microcystis flos-aquae*, MNs—*Microcystis natans*, MNv—*Microcystis novacekii*, MS—*Microcystis smithii*, MW- *Microcystis wesenbergii*; SS—separate cells; SL—cells of *Synechocystis aquatilis* type; TCs—colonies with transitional morphology; d—dominance (>25% of the total biomass); x—occurrence; r—rare occurrence in single colonies (for details, see the text of the paper).

**Table 2:** Sampling sites and their environmental parameters with types of found cyanotoxins Bulgarian waterbodies (WBs) in the period 21–25 June 2018 (after [4]).

WBN and IBW	SAN	Date	Alt	Latitude	Longitude	WT	pH	SD	CND	TDS	DO	TP	TN	CT
Res. Sinyata Reka (IBW1890)	SR1	21.06.18	317	42°28.1480’	24°42.217	27.4	9.72	0.5	470	305	9.36	25	4.8	MCs
	SR2	21.06.18	317	42°28.1473’	24°42.2175	26.7	9.36	0.6	468	306	9,21	27	4.3	
L. Vaya (IBW0191)	VA1	22.06.18	−2	42°30.5940’	27°22.075	26.9	9.65	0.25	2588	1682	12.51	13	5,4	CYN
	VA2	22.06.18	0	42°28.4540’	27°25.482	28.28	8.86	0.25	1183	768	11.94	11	3.7	
	VA3	23.06.18	6	42°29.1850’	27°26.531	23.7	9.5	0.25	1024	665	7.01	12	4.6	
Res. Mandra (IBW1720)	MN1	23.06.18	12	42°24.0463’	27°26.1120’	25.88	8.28	0.4	649	421	6.81	3	3.0	
	MN2	23.06.18	13	42°24.0670’	27°19.1310’	26.2	8.2	0.2	663	461	5.89	6	4.0	CYN
	MN3	23.06.18	9	42 26.1420’	27°26.5860’	24.9	8.48	0.3	639	415	7.91	4	3.3	
L. Uzungeren (IBW0710)	UZ	23.06.18	7	42°26.1782’	27°27.1998’	25.9	8.06	0.4	14.38	9351	7.83	5	2.8	
Res. Poroy (IBW3038)	PR	24.06.18	41	42°43.0190’	27°37.3160’	25.10	8.33	1.2	762	495	9.45	1	2.8	
Res. Aheloy (IBW3032)	AH	24.06.18	144	42°42.8230’	27°30.9740’	25.4	8.51	1.10	614	399	8.92	1	4.1	
L. Ezerets (IBW0233)	EZ	25.06.18	−2	43°35.2770’	28°33.2290’	26.4	8.35	TTB	1084	0	9.94	0.5	5.3	
L. Shabla (IBW0219)	SH	25.06.18	−2	43°33.8180’	28°34.1860’	27.1	8.46	TTB	1087	0706	9.97	0.1	5.1	
L. Durankulak (IBW0216)	DR1	25.06.18	6	43°40.3240’	28°32.0470’	24.03	8.54	1	1111	722	7.35	21	2.8	
	DR2	25.06.18	6	43°40.3340’	28°32.0220’	24.7	8.21	1	1094	711	7.79	20	4.0	MCs
	DR3	25.06.18	4	43°40.5300’	28°32.9930’	24.6	8.49	1	1075	698	6.19	24	3.9	MCs, SXT
	DR4	25.06.18	3	43°40.6950’	28°32.6000’	26.5	8.53	1	1087	706	9.6	20	3.2	

WBN—name of the water body; IBW—number in the Inventory of Bulgarian Wetlands [31]; Res—reservoir; L—lake; SAN—site abbreviation and number; Alt—altitude; WT—water temperature (°C), SD—Secchi depth (m); TTB—total transparent to bottom; CND—conductivity (µS); TDS—total dissolved solids (µg L^−1^); DO—oxygen concentration (mg L^−1^); TP—total phosphorus (mg L^−1^); TN—total nitrogen (mg L^−1^); CT—cyanotoxins, MCs—microcystins, CYN—cylindrospermopsin, SXT—saxitoxins.
